# Supplementary figures and images for: Valproic acid stimulates myogenesis in pluripotent stem cell-derived mesodermal progenitors in a NOTCH-dependent manner
Source: Cell Death Dis. 2021 Jul 5;12(7):677. doi: 10.1038/s41419-021-03936-w (PMC8257578; doi:10.1038/s41419-021-03936-w)

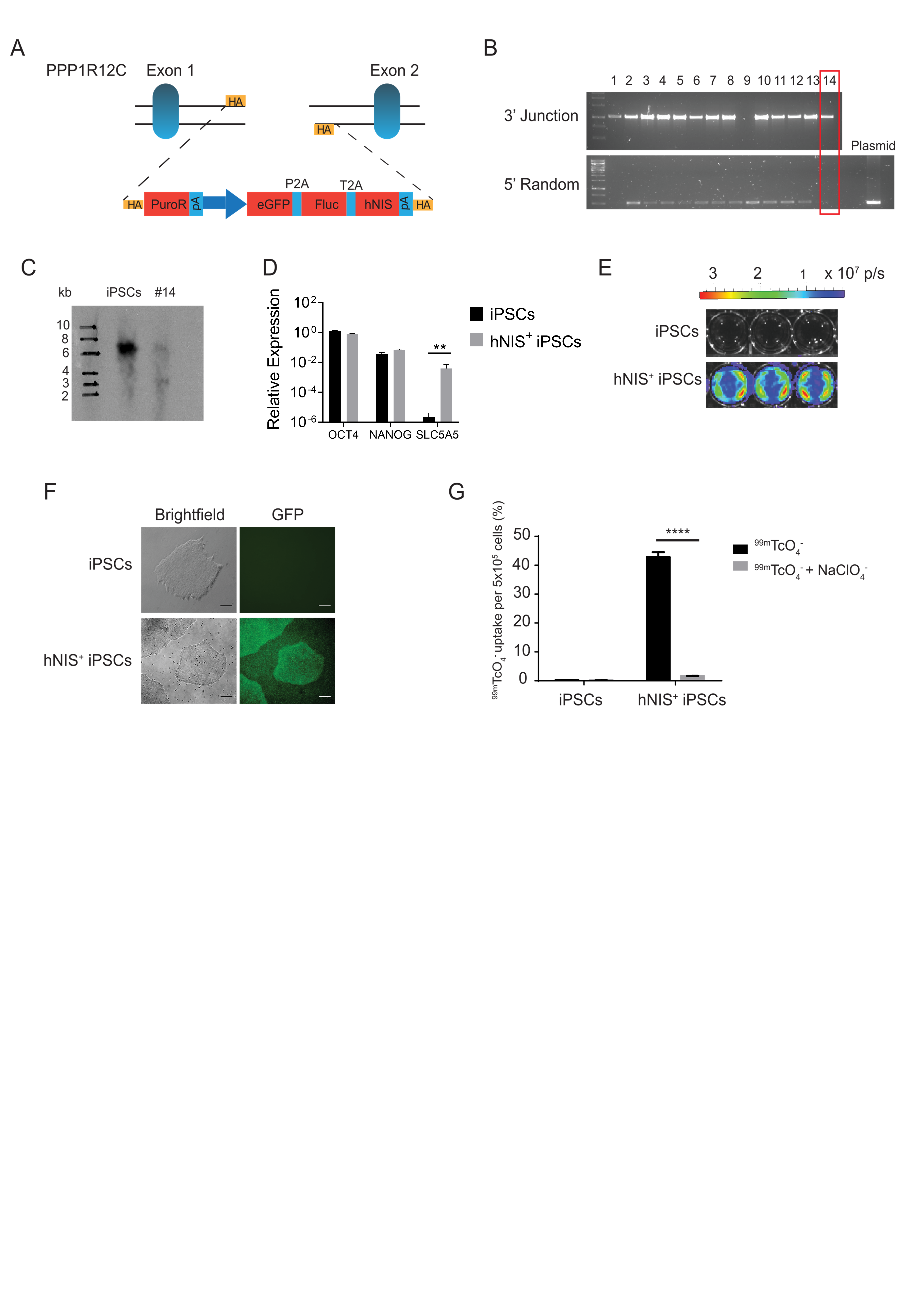

Supplement: Supplementary file 1 — Supplementary Figure 1 [file 41419_2021_3936_MOESM1_ESM.png]

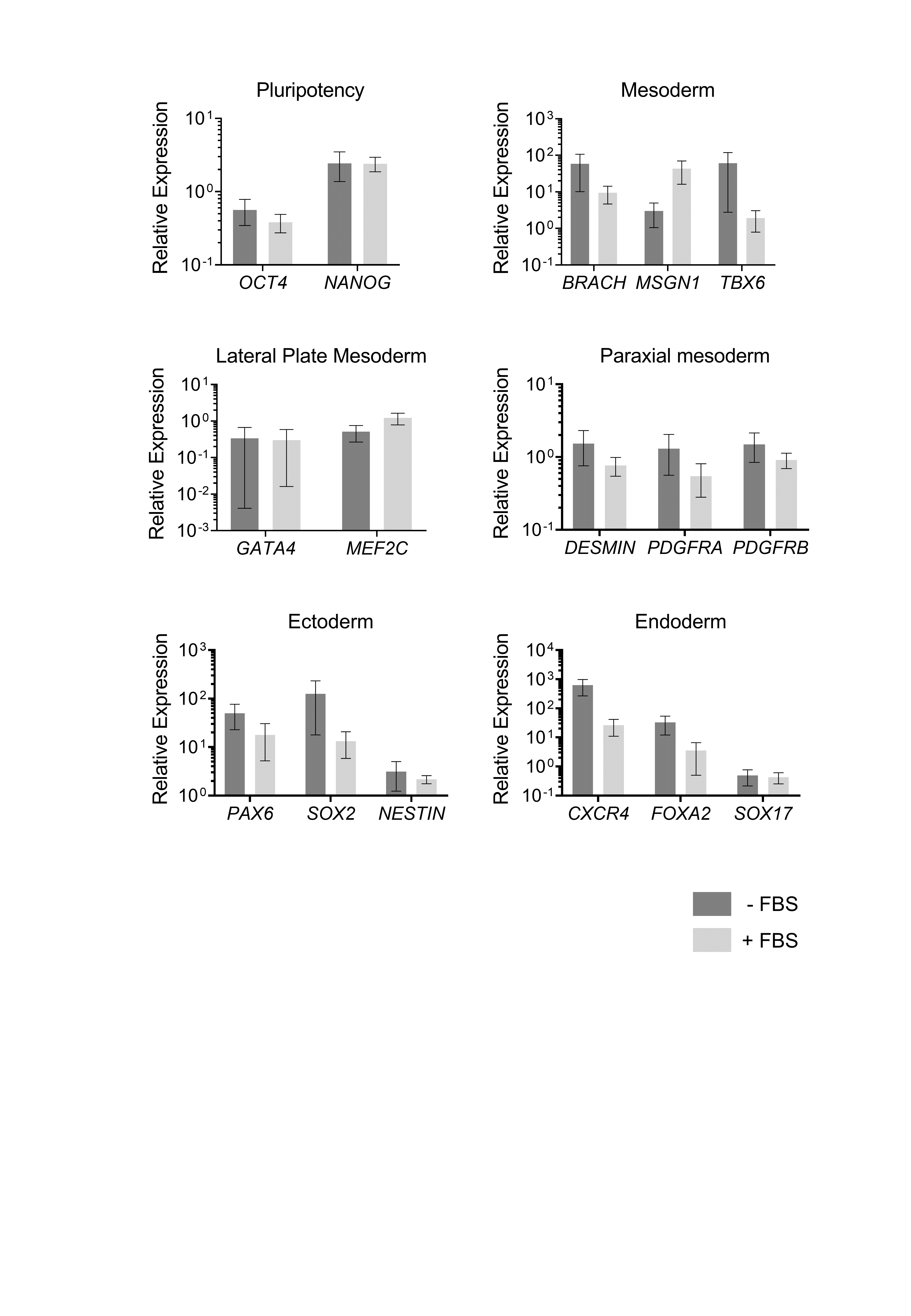

Supplement: Supplementary file 2 — Supplementary Figure 2 [file 41419_2021_3936_MOESM2_ESM.png]

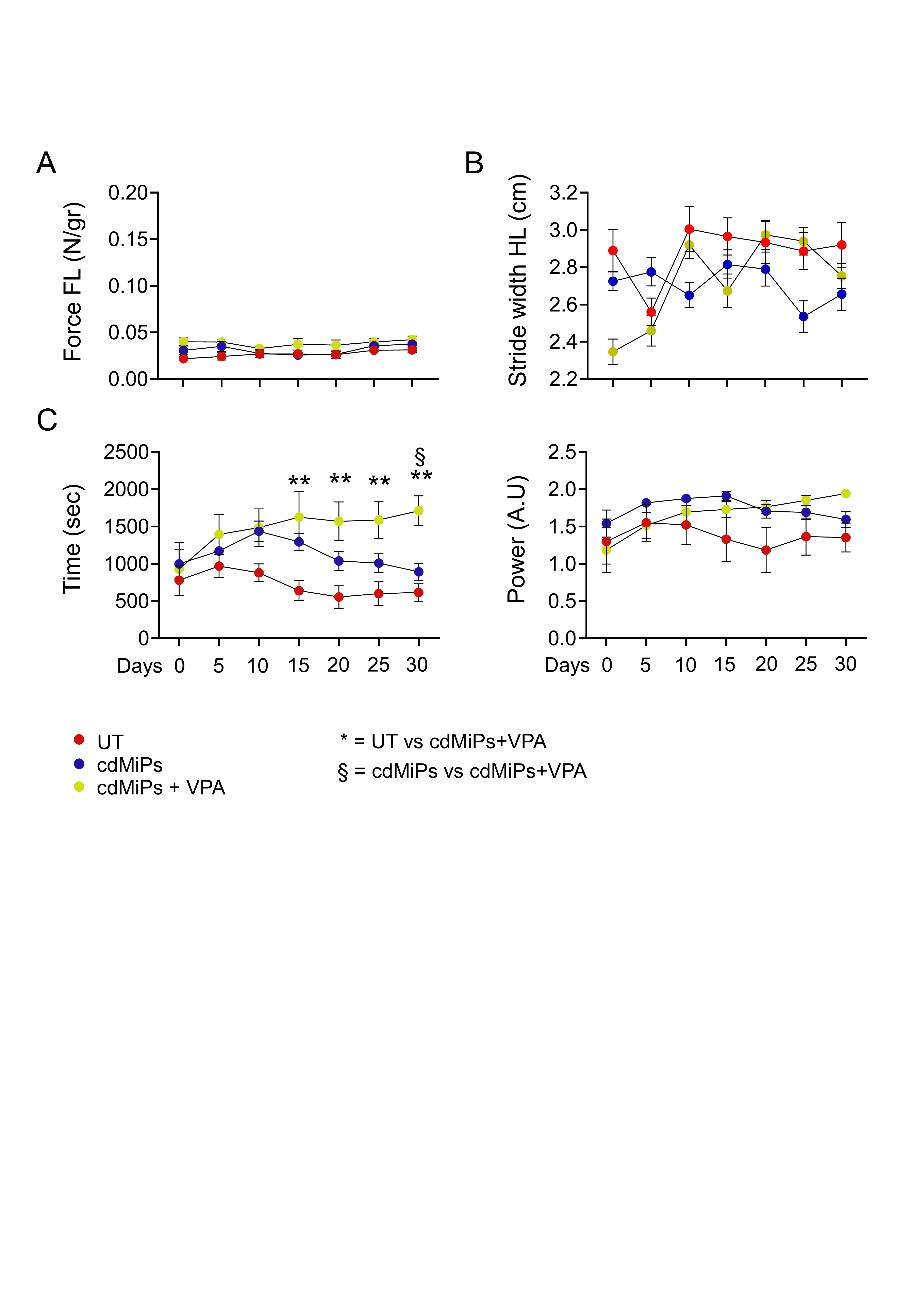

Supplement: Supplementary file 3 — Supplementary Figure 3 [file 41419_2021_3936_MOESM3_ESM.png]

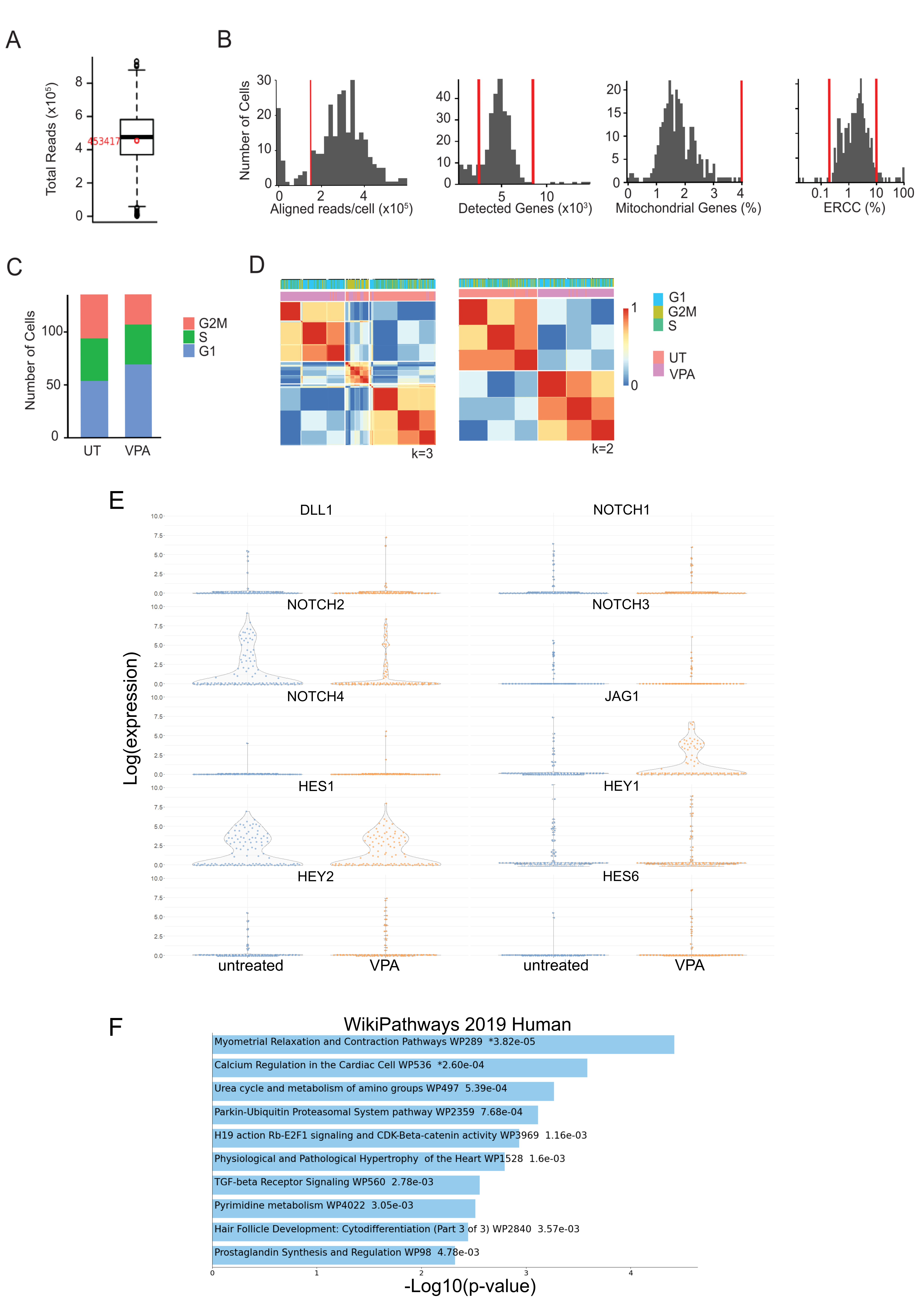

Supplement: Supplementary file 4 — Supplementary Figure 4 [file 41419_2021_3936_MOESM4_ESM.png]
