# Supplementary material for: Valproic acid stimulates myogenesis in pluripotent stem cell-derived mesodermal progenitors in a NOTCH-dependent manner
Source: Cell Death Dis. 2021 Jul 5;12(7):677. doi: 10.1038/s41419-021-03936-w (PMC8257578; doi:10.1038/s41419-021-03936-w)
Supplement: Supplementary file 5 — Suplementary TABLES [file 41419_2021_3936_MOESM5_ESM.docx]

**Supplementary Table 1. List of RT-PCR primers from 5’-3’.**

| **Gene** | **Forward primer** | **Reverse primer** |
| --- | --- | --- |
| ***OCT4*** | CGAGCAATTTGCCAGCTCCTGAA | GCCCAGCTTACACATGTTCTTGA |
| ***NANOG*** | GATTTGTGGGCCTGAAGAAA | AAGTGGGTTGTTTGCCTTTG |
| ***BRACH*** | ACCCAGTTCATAGCGGTGAC | AAGCTTTTGCAAATGGATTG |
| ***MSGN1*** | CTGCACACCCTCCGGAATTA | AGGAGGTCTGTGAGTTCCCC |
| ***TBX6*** | GTGTCTTTCCATCGTGTCAAGC | TATGCGGGGTTGGTACTTGTG |
| ***DESMIN*** | GAAGCTGCTGGAGGGAGAG | ATGGACCTCAGAACCCCTTT |
| ***PDGFRA*** | TGGCAGTACCCCATGTCTGAA | CCAAGACCGTCACAAAAAGGC |
| ***PDGFRB*** | GCTCACCATCATCTCCCTTATC | CTCACAGACTCAATCACCTTCC |
| ***PAX6*** | AACGATAACATACCAAGCGTGT | GGTCTGCCCGTTCAACATC |
| ***SOX2*** | TGGCGAACCATCTCTGTGGT | CCAACGGTGTCAACCTGCAT |
| ***NESTIN*** | TCAGCTTTCAGGACCCCAAG | TGGGAGCAAAGATCCAAGACG |
| ***CXCR4*** | GGTGGTCTATGTTGGCGTCT | TGGAGTGTGACAGCTTGGAG |
| ***SOX17*** | GTGGACCGCACGGAATTTG | GGAGATTCACACCGGAGTCA |
| ***FOXA2*** | AGGAGGAAAACGGGAAAGAA | GGTGCTTGAAGAAGCAGGAG |
| ***GATA4*** | TCATCTCACTACGGGCACAG | GGGAAGAGGGAAGATTACGC |
| ***MEF2C*** | CAGACATCGTGGAGACGTTGA | GCTGTGACCTACGGAATCGT |
| ***SLC5A*** | CTCCCTGCTAACGACTCCAG | AGGTCCCACCACAACAATC |
| ***RLP13A*** | CCTGGAGGAGAAGAGGAAAGAGA | TGGAGGACCTCTGTGTATTTGTCAA |
| ***GAPDH*** | TCAAGAAGGTGGTGAAGCAGG | ACCAGGAAATGAGCTTGACAAA |
| ***ACTB*** | GCCAAGTCGGTAGTCCTTATG | CCCAGCAGAGATTTGAGTTCTA |
